# Underwater Sensor Network Redeployment Algorithm Based on Wolf Search

**DOI:** 10.3390/s16101754

**Published:** 2016-10-21

**Authors:** Peng Jiang, Yang Feng, Feng Wu

**Affiliations:** College of Automation, Hangzhou Dianzi University, Hangzhou 310018, China; yangfengfy@126.com (Y.F.); fengwu@hdu.edu.cn (F.W.)

**Keywords:** underwater sensor networks, 3D node deployment, coverage, wolf search

## Abstract

This study addresses the optimization of node redeployment coverage in underwater wireless sensor networks. Given that nodes could easily become invalid under a poor environment and the large scale of underwater wireless sensor networks, an underwater sensor network redeployment algorithm was developed based on wolf search. This study is to apply the wolf search algorithm combined with crowded degree control in the deployment of underwater wireless sensor networks. The proposed algorithm uses nodes to ensure coverage of the events, and it avoids the prematurity of the nodes. The algorithm has good coverage effects. In addition, considering that obstacles exist in the underwater environment, nodes are prevented from being invalid by imitating the mechanism of avoiding predators. Thus, the energy consumption of the network is reduced. Comparative analysis shows that the algorithm is simple and effective in wireless sensor network deployment. Compared with the optimized artificial fish swarm algorithm, the proposed algorithm exhibits advantages in network coverage, energy conservation, and obstacle avoidance.

## 1. Introduction

Two-thirds of the Earth surface is covered with water. Underwater wireless sensor networks (UWSNs) were developed to meet the need for environmental data collection, underwater tactic monitoring, environmental monitoring, and others [1,2,3,4]. UWSN coverage control as the foundation of data aggregation, routing protocol design, positioning and tracking [5,6,7,8] has a direct effect on network coverage rate, quality of service, and network lifetime. It has become one of the popular topics in the information field. Practical 3D coverage control algorithms need to consider fully the actual situation of the underwater node distribution. It also needs to adjust the position according to the monitor target and change in the water environment to optimize the coverage of the entire network. Compared with terrestrial sensor networks, UWSNs are mainly characterized by acoustic communication, signal delay and attenuation, sparse distribution of nodes, self-organization capability, and easy changing of the network topology under the influence of water flow and aquatic organisms [9]. In the large-scale deployment of UWSNs, underwater nodes are difficult to replace when batteries fail. All of these conditions are significant challenges in the process of UWSN redeployment.

In the past, UWSN redeployment algorithms were mainly based on graph theory, cube lattice category, virtual force and swarm intelligence optimization, and other related ways [10,11,12,13,14,15]. The first three types of redeployment algorithms are relatively complex and unsuitable for large-scale underwater environmental problems. The swarm intelligence optimization algorithm [16] is generally much faster than traditional optimization algorithms in terms of optimal solution search and simple calculation. The advantage is that it does not provide a global model and centralized control. The algorithm possesses strong applicability and generality [17]; therefore, it is preferred by scholars in the field of UWSNs [18,19,20,21,22]. Huazheng Du et al. [19] proposed an algorithm for underwater sensor networks based on particle swarm optimization. The algorithm solves the problem of node deployment, and it has the advantage of high convergence speed. Lyer et al. [20] proposed a positioning and deployment scheme for UWSNs based on the genetic algorithm by using optimization techniques. Their main objective was to achieve a coverage area of interest with the least number of nodes but easily falls into a local optimum of the considered objective function.

Yiyue et al. [21] proposed a redeployment algorithm for UWSNs based on artificial fish (OAFSA). The algorithm is an improved version of the traditional fish school algorithm. A dynamic threshold was added to foraging behavior. Naturally, the coverage rate was improved. However, the latter easily falls into a local optimum of the considered objective function. Redeployment algorithms based on swarm intelligence optimization are intuitive and easy to describe and prove. Swarm intelligence optimization algorithms exhibit common problems of poor robustness in the late stage, low convergence speed, and ease of falling into a local optimum of the considered objective function. Therefore, the search for a new swarm intelligence optimization algorithm that exhibits excellent performance in the field of UWSNs is highly significant. After the initial deployment, nodes will move freely according to the specific algorithm again，and they will finally reach coverage demand. The paper uses this redeployment.

The UWSN deployment algorithm of obstacle avoidance is inspired by the predator avoidance of the wolf search algorithm. Existing redeployment algorithms generally disregard the presence of obstacles in the underwater environment. However, in reality, obstacles hinder the movement of nodes. For example, a hidden reef in a lake or ocean, swimming fish, and dense grasses are obstacles that not only affect node movement but also interfere with the communication among nodes. Many obstacles exist in actual UWSN deployment. These obstacles greatly affect the network topology. Therefore, this study considers obstacles as an important factor in the process of UWSN node deployment.

To address the obstacles in actual water environments and the demand for simple calculation, this paper presents the underwater sensor network redeployment algorithm based on wolf search (RAWS) to obtain good underwater network coverage. The wolf search algorithm has two advantages over other swarm intelligence algorithms. These two advantages are independent search and predator avoidance. After the network model and underwater sensing node model are decided, it combines with the independent search characteristics of the wolf search algorithm. Each node can implement independent search in the deployment process. Any two nodes will not move in the same direction unless a common coverage target exists. They can rapidly search, and they have global optimization ability. Different from other swarm intelligence optimization algorithms, the proposed algorithm eliminates a wide range of internal communication among individuals and reduces computational complexity. Furthermore, it sets the escape mechanism in the process of node movement, and thus it avoids the aquatic organism obstacles. It allows the algorithm to jump out of the local optimal solution to maintain the diversity of the latter part of the solution space. The simulation results show that the distributed redeployment algorithm can effectively increase network coverage and avoid node failure resulting from external force.

The rest of this paper is organized as follows: in Section 2, the related works are introduced; in Section 3, the three models of UWSNs and related definitions are described; in Section 4, the problem is analyzed, and the principle and the progress of the RAWS algorithm are implemented. In Section 5, the description of algorithm simulation and the detailed analysis of the simulation results are discussed. Finally, in Section 6, the conclusions and future work of our study are drawn.

## 2. Related Works

The research of the UWSN (Underwater wireless sensor network) node deployment algorithm mainly focused on the static deployment and dynamic deployment. Static deployment includes determining deployment and random deployment. They mainly used no mobile node of the network [23,24]. The random deployment will lead to a lot of redundant nodes. And it uses aircraft to put nodes in the place that human is not convenient to arrive. Static deployment is that the sensor nodes’ locations are known. To cover the monitoring area, the disadvantage is that the deployment efficiency is low. Dynamic deployment can solve the two types of deployment listed above [25,26]. The dynamic deployment can be divided into two types. One of them is the mobile limited dynamic deployment. It means that part of the nodes move limitedly, and they are only allowed to move in one direction. For example, it includes depth regulation deployment. The other one of them is the freedom dynamic deployment. It means that nodes have the ability to move freely, so they can plan a trajectory that is not constrained. Dynamic deployment according to the specific algorithm can automatically adjust the position in UWSNs, until it reaches the network coverage requirements [27]. Therefore, in order to achieve a better effect, we adopt a mobile free node deployment algorithm to solve the problem of underwater node deployment.

Compared with the existing algorithms, the proposed RAWS (redeployment algorithm based on wolf search) algorithm contributions are as follows:

(1) the underwater deployment scenarios consider real obstacle factors;
(2) in order to achieve a better effect, when faced with obstacles, the nodes can avoid obstacles;
(3) the obstacle avoidance mechanism avoids node failure due to external force. From a certain extent, it reduces the network energy consumption and guarantees the stability of network topology.

## 3. System Model

### 3.1. Network Model

We assume that *N* sensor nodes are randomly and uniformly placed in a target water area and sink nodes are randomly distributed on the surface of the water. Once a node is in the water, it will anchor itself. Each node is capable of communication, perception, and mobility. The symbol *s_i_* is used to denote the *i*-th node position, and the corresponding node set is $S=\{s_{1},s_{2},\ldots ,s_{n}\}$. The following assumptions are established.

(1) In addition to the sink nodes, all nodes in the network have the same communication and sensing radii.
(2) When the network is initialized, the nodes are distributed randomly in the 3D monitoring space, and the nodes can sense their own location information and their neighbors.
(3) The obstacles (irregular and polyhedral) are distributed randomly in the underwater 3D monitoring space. If a node hits an underwater obstacle in the process of moving, node communication will break down.

### 3.2. Node Perception Model

Network coverage rate reflects the coverage degree of the underwater sensor network covering the monitoring area or targets. The probabilistic sensing model was utilized in this study to calculate the network coverage rate. The sensing range of the node is a spherical region with the node as the center and radius r. Among them, $p_{j}$ $\left(a_{j},b_{j},c_{j}\right)$ expresses the position of the *j*-th monitored target, and $d(s_{i},p_{j})$ express the distance between nodes and the monitoring target. The formula is defined as follows:

(1) $$d(s_{i},p_{j})=\sqrt{{\left(x_{i}-a_{j}\right)}^{2}+{\left(y_{i}-b_{j}\right)}^{2}+{\left(z_{i}-c_{j}\right)}^{2}}$$

$f(s_{i},p_{j})$ expresses the probability that the *i*-th node ensures coverage for *j*-th event, and is formulated as follows:

(2) $$f(s_{i},p_{j})=\{\begin{array}{c} 0,\;r+Re\;\leq \;d\left(s_{i},p_{j}\right)\\ e^{-\alpha \lambda^{\beta }},\;\;r-Re\;\;<\;d\left(s_{i},p_{j}\right)\;<\;r\;+\;Re\\ 1,\;r-Re\;\geq \;d\left(s_{i},p_{j}\right) \end{array}$$

where *Re* is an uncertain factor in the measurement range of underwater nodes and satisfies 0 < *Re* < *r*. α and β are related to the parameters of the measurement equipment. λ is the input parameter defined as follows:

(3) $$\lambda =\;d\left(s_{i},p_{j}\right)-\left(Rs-Re\right)$$

To calculate the coverage rate of UWSNs, the probability of monitoring should be greater than 0. The ratio of the value to the total target monitoring points (*n*) is the network coverage rate (*C*). The formulation is as follows:

(4) $$C=(\sum_{1}^{n}f\left(p_{j},s_{i}\right)\neq 0)/n$$

where $f\left(p_{j},s_{i}\right)\neq 0$ denotes the *i-*th node covering the *j-*th event. It means that the *i-*th node is covered. The $\sum_{1}^{n}f\left(p_{j},s_{i}\right)\;\neq \;0$ denotes the covered nodes’ total number. The *n* denotes the nodes’ total number. Thus, we define that the coverage rate *C* is the ratio of the covered nodes’ total number and the nodes’ total number.

**Figure 1:**
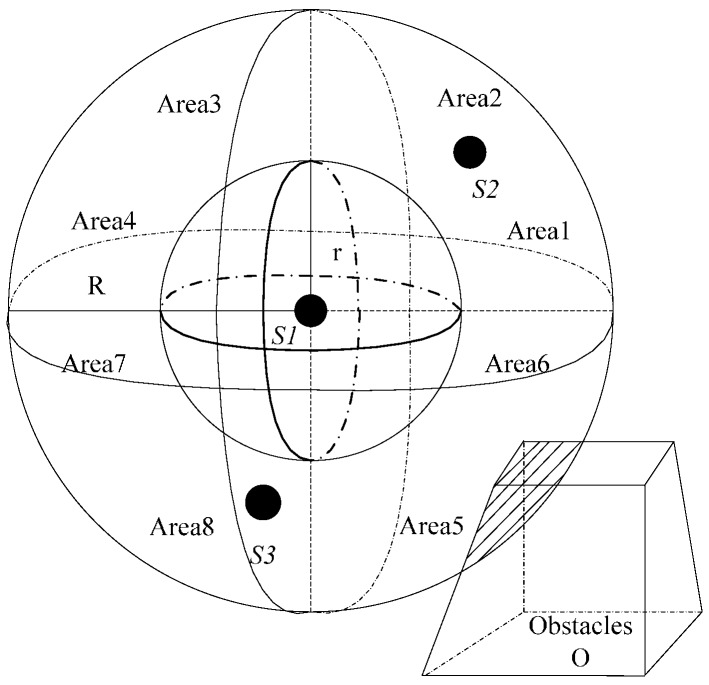
Division map of an underwater node communication area.

To calculate node coverage, it must consider the node density and obstacle density. As shown in Figure 1, the scope of node communication is a sphere, and it is equally divided into *k* districts. When the *k* is large, the density calculation is more accurate. The irregular polyhedron represents obstacles in Figure 1. The node communication radius is *R*. The perceived radius is *r*. Regions 1 to 4 distribute in the above hemisphere from front the back and from left to right, and the regions 5 to 8 distribute in the below hemisphere in the same way. The node density of S1 is as follows:

(5) $$D=N_{A}/N_{t},$$

where *D* represents the node density, *N_A_* is the number of nodes in the *S1* region, and *N_t_* represents the minimum number of nodes to cover the entire partition. *N_t_* depends on the sensing and communication radii of the node. It can be calculated according to the formula as follows:

(6) $$N_{t}=C\frac{K_{m}}{V_{ball}}=3\times (1-\frac{3V_{z}}{4\pi r^{3}})\cdot \frac{K_{m}}{4\pi r^{3}},$$

where *C* represents the effective coverage rate, *K_m_* represents the volume of a single partition of the node, and *V_z_* represents the volume of the overlapping area. In the same manner, the density of the obstacle is defined as follows:

(7) $$D_{o}=\frac{O_{m}}{K_{m}},$$

where *D_o_* is the obstacle density and *O_m_* is the volume of the obstacles detected by the node.

First of all, the node perception model has been described. It can better reflect the monitoring process of node coverage targets. Then, the density models of obstacles and nodes have been shown. The RAWS algorithm described below will involve the problem of the relationship between nodes and obstacles. Thus, using the three-dimensional model displays intuitively better.

### 3.3. Underwater Energy Consumption Model

The RAWS algorithm should not only consider the coverage problem in the node deployment process, but also consider the energy consumption problem in the mobile process of nodes. Because once some nodes run out of energy consumption, the underwater nodes are not convenient for replacing batteries. Then, the coverage effect will decrease. So we can adjust timely the nodes according to the energy consumption situation. For the underwater deployment algorithm, the choice of the energy consumption model is crucial [28].

Underwater sensor networks adopt special underwater acoustic communication, and the energy consumption model of an underwater network is based on the sound wave. This study used this model to consider the energy consumption of the underwater network. *E_tx_*(*d*) expresses the energy that nodes send data as follows:

(8) $$E_{tx}(d)=P_{r}\times T_{p}\times A(d),$$

where *d* is the data transmission distance, *T_p_* is the data transmission time, and *P_r_* are the minimum power packets that can be received. When the transmission distance of *A*(*d*) is *d*, the underwater acoustic signal attenuation model *A*(*d*)is provided by

(9) $$A(d)=d^{\lambda }\alpha^{d},$$

where *λ* is the energy diffusion factor (spherical diffusion to 2) and parameter *α =* 10*^a^*^(*f*)*/*10^, as determined by absorption coefficient *a*(*f*) [29]. The formulation is as follows:

(10) $$a(f)=0.11\frac{10^{-3}f^{2}}{1+f^{2}}+44\frac{10^{-3}f^{2}}{4100+f^{2}}+2.75\times 10^{-7}f^{2}+3\times 10^{-6},$$

where *f* is the carrier frequency (unit: kHz). The unit of the absorption coefficient is dB/m. 

## 4. RAWS Algorithm

The wolf search algorithm with ephemeral memory (WSA) is a new type of swarm intelligence optimization algorithm proposed by Tang et al. [30] in 2012. The algorithm is based on the simulation of preying and escaping from predators. Wolves generally exhibit three typical behaviors: preying initiatively, preying passively, and escaping. The swarm behavior of this intelligent optimization algorithm is different from that of others. Each wolf has an independent search capability, thus increasing the diversity of the search space. The algorithm has a mechanism to avoid predators, which other swarm intelligence optimization algorithms do not have. Thus, wolves avoid falling into the local optima in the search process.

### 4.1. RAWS Basic Principle

**Figure 2:**
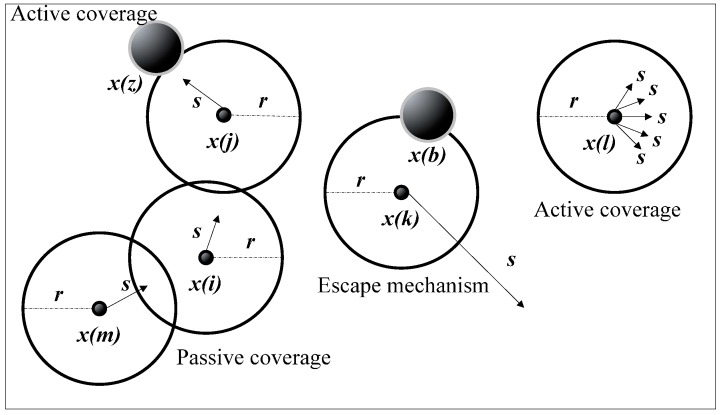
Basic principle diagram of RAWS (redeployment algorithm based on wolf search).

The objective of this study is to deploy sensor nodes in a certain 3D underwater space. When the UWSN node was deployed randomly, the redeployment scheme was designed according to the WSA algorithm, as shown in Figure 2.

Active coverage: When node *x*(*j*) detects the presence of coverage target *x*(*z*) in the sensing range, the node moves to the target with a certain step. In the absence of a common target, any two nodes will not move in the same direction.

Passive coverage: It uses distributed coverage when it doesn’t have a target in the sensing range. If node *x*(*l*) does not have a neighbor node with a hop, then node *x*(*l*) can move in any direction with a certain step. If node *x*(*i*) has a one hop neighbor node and the neighbor node *x*(*j*) has more targets of monitoring points than node *x*(*i*), then node *x*(*i*) moves along the direction of the best neighbor node *x*(*j*) in a certain step.

Escape mechanism: If node *x*(*k*) is aware of obstacles of *x*(*b*), then it moves in a random direction with a large sensing radius of the step size to avoid obstacles. This mechanism can prevent the algorithm from falling into a local optimum of the considered objective function solution.

### 4.2. RAWS Algorithm Description and Process

In combination with the basic theory of the RAWS algorithm, the underwater sensor node redeployment scheme was implemented in a distributed manner.

As shown in Section 4.2, *N*_event_(*s_i_*) is the target monitoring points of node B. The formulation is as follows:

(11) $$N_{\mathrm{event}}\left(s_{i}\right)=\sum_{1}^{n}f\left(p_{j},s_{i}\right)\neq 0.$$

The set of neighbor nodes is *Z*(*s_i_*), and the number of neighbor nodes is *N*_nei_(*s_i_*):

(12) $$Z\left(s_{i}\right)=\{s_{z}|d\left(s_{i},s_{z}\right)\leq Rc,z=1,2,3,\;\ldots \ldots ,\;n\},$$

(13) $$N_{\mathrm{nei}}\left(s_{i}\right)=\mathrm{card}\left(K\left(s_{i}\right)\right).$$

Initially, *n* sensor nodes were randomly deployed in the 3D underwater monitoring space. The initial random coverage was calculated, and then nodes were redeployed according to the RAWS algorithm.

Step 1: Target monitoring points exist around node *s_i_*, and the nodes are not too crowded (node concentration D is reasonable). That is, when *N*_event_(*s_i_*) ≠ 0, the node covers the target monitoring point and does not move.

Step 2: When no target monitoring point exists around node *s_i_*, that is, *N*_event_(*s_i_*) = 0, two situations exist.

Step 2.1: If one hop neighbor node exists around node A, that is, *N*_event_(*s_i_*) = 0,the nodes will be randomly moved to the new location of *P*(*i*)_1_ in any direction to maintain the diversity of the objective function value in the optimization process (shown in Equation (14)). Assuming that *s_i_* perceives the obstacles in the process of moving, the node will avoid the obstacles and jump to *P*(*i*)_2_ with the greater distance than the node sensing radius. This escape mechanism can not only make the node move safely but also prevent the algorithm from falling into a local optimum of the considered objective function solution:

(14) $$P{\left(i\right)}_{1}=P\left(i\right)+\partial \cdot r\cdot \mathrm{rand}()\cdot L,$$

where *P*(*i*) is the initial location of the node, *P*(*i*)_1_ is the new position after the node has moved, *V* represents any unit vector, and *∂* is the speed rate. With the increase in distance, the movement speed decreases, as shown in Equation (15). *Rs* is the node sensing radius. rand()∙*L* represents the direction of random movement of nodes, as shown in Equation (16):

(15) $$\partial \left(r\right)=\partial_{0}e^{-r^{2}}$$

(16) $$L=\frac{\left(P\left(i\right)-V\right)}{\left|P\left(i\right)-V\right|}$$

where *s* represents the moving step size that is less than node sensing radius *Rs*. escape()∙*L* is the location for random escape. It is greater than the node sensing radius and less than half of the monitoring area. If a node moves beyond the monitoring area, the node will move in the reverse direction of the escape formula, as shown in Equation (17).

(17) $$P{\left(i\right)}_{2}=P{\left(i\right)}_{1}\pm \partial \cdot s\cdot \mathrm{escape}()\cdot L$$

Step 2.2: If other neighbor nodes exist around node *s_i_*, that is, *N*_event_(*s_i_*) ≠ 0, and node *s_i_* will find the neighbor node with the most coverage monitoring points. This neighbor node is denoted as the best neighbor node *s_p_* (Equation (18)). Then, node *s_i_* moves to the optimal neighbor node in one step, as shown in Equation (19). Assuming that the node encounters obstacles in the process of moving, and the node escapes from these obstacles, as shown in Equation (17):

(18) $$s_{p}=\max\left\{N_{\mathrm{even}t}\left(Z(s_{i}\right)\right\},$$

(19) $$P{\left(i\right)}_{1}=P(i)+\partial \cdot Rs\cdot L\left(i\to p\right),$$

(20) $$L\left(i\to p\right)=\frac{\left(P\left(i\right)-P\left(p\right)\right)}{\left|P\left(i\right)-P\left(p\right)\right|},$$

where L(i→p) is the unit direction vector of node B to node A, and P(p) is the coordinate position of node *s_p._*

**Figure 3:**
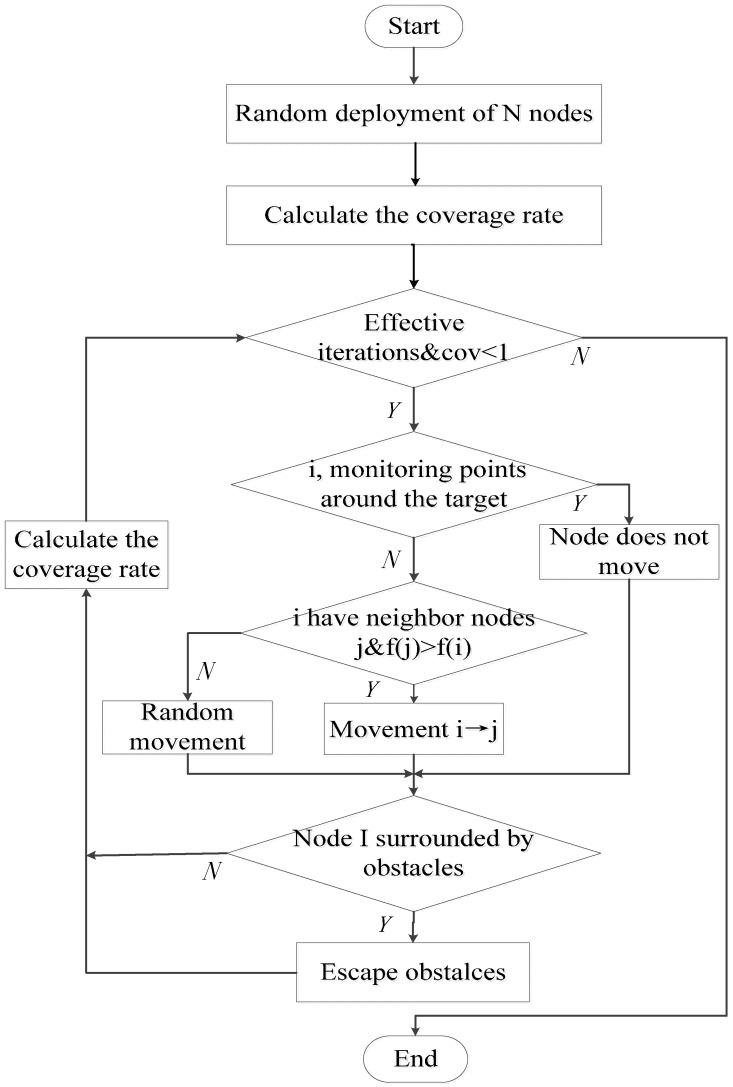
Flowchart of the RAWS algorithm.

Node deployment of the UWSN is thus completed. The flowchart of the RAWS algorithm is shown in Figure 3.

Because each node has ability to search independently in the process of node redeployment, the RAWS algorithm has a strong global optimization capability. This capability reduces internal exchange and movement. The algorithm also adopts congestion control and has effective node coverage. Finally, in the process of escape, it effectively avoids node failure because of external forces. Thus, the algorithm can jump out of the local optimal solution, and the diversity of the local optimal solution is increased. Therefore, the RAWS algorithm has good optimization performance.

## 5. Simulation and Performance Analysis

### 5.1. Introduction of an Algorithm for Comparison and an Evaluation Indicator

The OAFSA algorithm is a typical swarm intelligence algorithm. The OAFSA algorithm and the RAWS algorithm have a common characteristic of events being perceivable in the visual range. Therefore, to evaluate the performance of the RAWS algorithm, the OAFSA algorithm was selected for a comparison algorithm. Network coverage was used as the performance evaluation indicator. The OAFSA algorithm and the RAWS algorithm were simulated and analyzed.

The RAWS algorithm has several advantages over the OAFSA algorithm:

(1) each node is independent in the process of the search event. Expanding the search range reduces the internal node mobility and communication;
(2) if a node encounters obstacles in the process of moving, it will avoid them in time and jump out of the local optimal situation;
(3) the algorithm is combined with congestion control, which is conducive to node coverage.

### 5.2. Simulation Results and Analysis

The UWSN node deployment process was simulated with Matlab (R2011b, MathWorks, Natick, MA, USA) based on the background of Xixi Wetland water environment monitoring. The underwater monitoring range was set to 200 × 200 × 200 m^3^, and 20 obstacles were randomly distributed in the water. Initially, 100 nodes were randomly distributed in the monitored water area, and 200 target monitoring points were randomly distributed in the wetland water environment. In the Matlab simulation environment, the process of node deployment and optimization were simulated to verify the performance of the RAWS algorithm.

The meaning and value of the simulation parameters are shown in Table 1.

**Figure 4:**
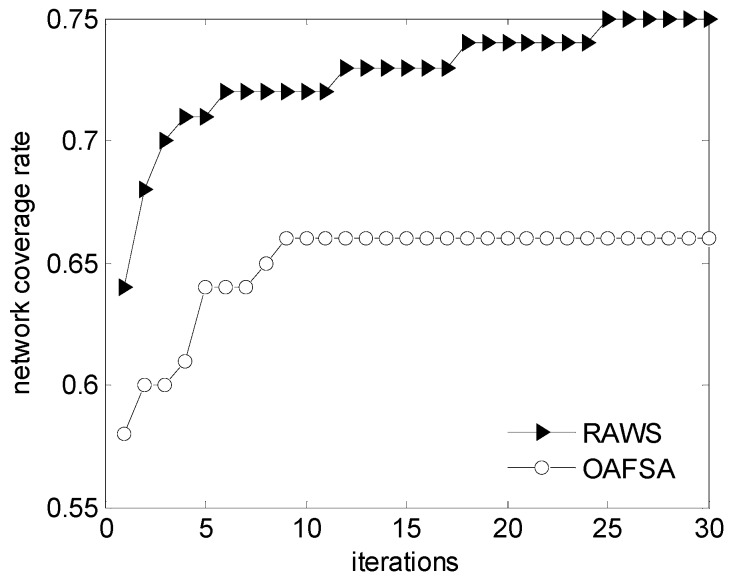
Comparison of network coverage rate at varying iterations.

**Figure 5:**
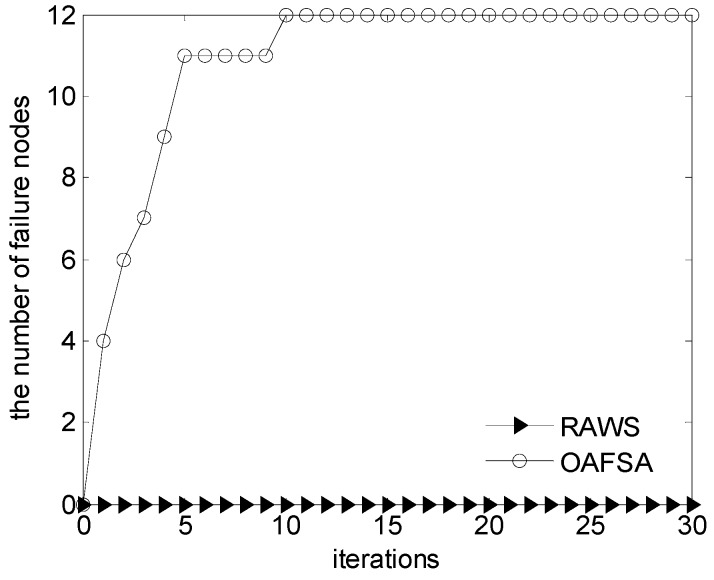
Comparison of the number of ineffective nodes at varying iterations.

Figure 4 shows the network coverage rate of the OAFSA and RAWS algorithms with the change in the number of iterations. The graph shows that the network coverage rate of the OAFSA algorithm stabilizes after 10 iterations. However, the RAWS algorithm stops the iteration after 25 times. The nodes will continue to jump out of the local optimum and find the global optimal solution because the nodes in the RAWS algorithm will not fail in the process of moving. Some of the nodes in the OAFSA algorithm encounter obstacles in each iteration. According to the simulation data (Figure 5), when all iterations are completed, the 12 nodes have been failed. Therefore, the OAFSA network coverage rate increases 66% and no longer changes. The RAWS algorithm finally makes the network coverage rate reach 75%. At the initial stage of the iteration, a node can avoid obstacles when it judges the surrounding obstacles. It can also avoid the local optimal value and improve the network coverage. After 11 iterations, the nodes are still searching for the global optimal solution, and the coverage rate is improved by a small margin. The search stops after 25 iterations. This result shows that the network coverage rate of the RAWS algorithm is better than that of the OAFSA algorithm with the same number of iterations in the network.

**Figure 6:**
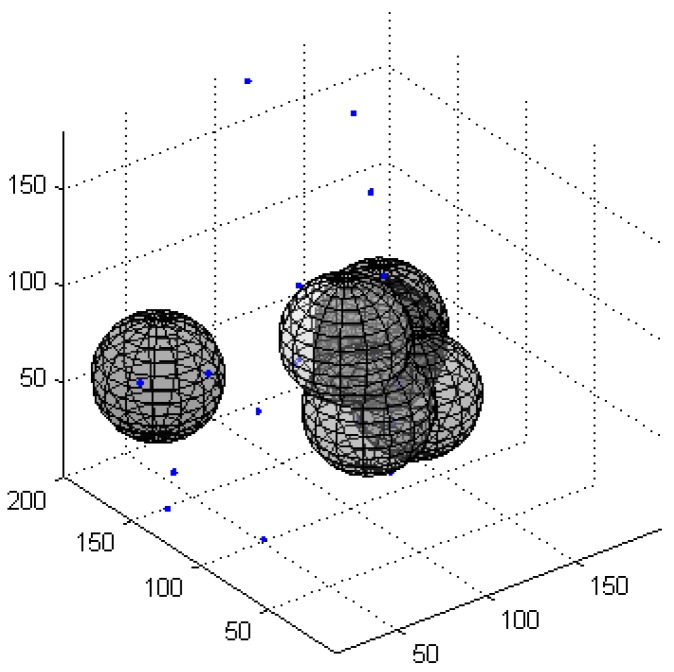
Layout of ineffective nodes in the second iteration.

**Figure 7:**
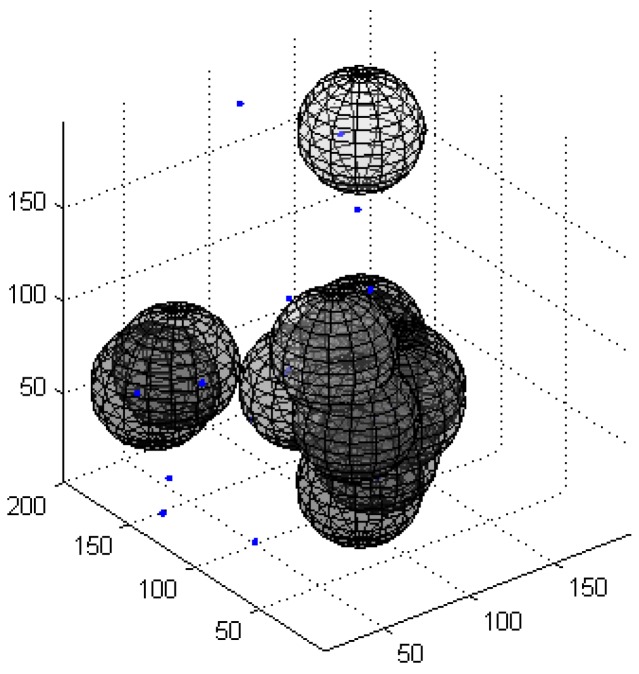
Layout of ineffective nodes in the tenth iteration.

Figure 5 presents the OAFSA and RAWS algorithms in the iteration process with the number of failure nodes. Figure 6 and Figure 7 show the 3D distribution of the invalid nodes of the OAFSA algorithm with two and 10 iterations, respectively. As shown in Figure 6, the nodes can avoid obstacles in time and do not experience node failure in the iteration process of the RAWS algorithm. In the early stage of the iteration process of the OAFSA algorithm, several nodes encounter obstacles. In second iteration, six invalid nodes exist. When the number of iterations reach 10, the nodes cease moving because the UWSN has reached the maximum coverage, and the number of failed nodes increases to 12 (Figure 7). The simulation results show that the RAWS algorithm can effectively avoid obstacles in water and prevent node failure resulting from external force. Thus, the stability of the underwater wireless sensor network topology is ensured.

**Figure 8:**
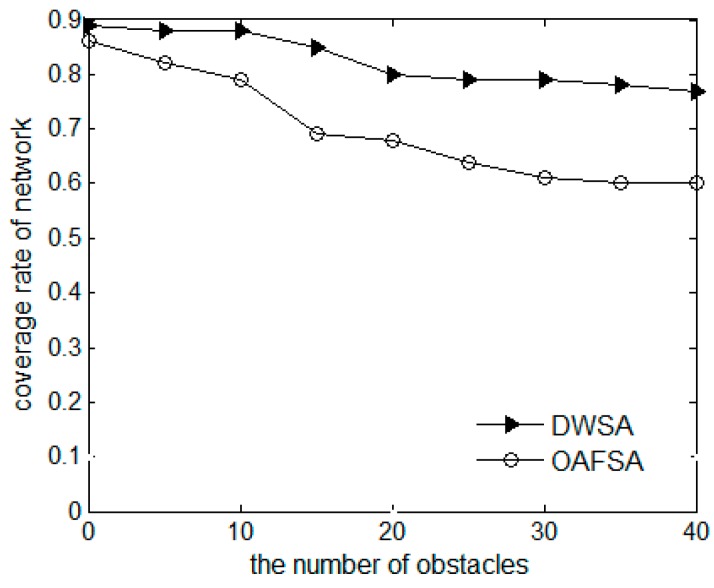
Comparison of network coverage rate at varying numbers of obstacles.

Figure 8 presents a comparison chart of the network coverage rate of the OAFSA and RAWS algorithms. Regardless of the amount of obstacles in the water, because the individual search of each node is independent, the RAWS algorithm can always maintain a relatively high coverage rate in the process of moving. When the optimal solution is found, only one hop neighbor node moves, which shows the advantage of the distributed algorithm over the large-scale aggregation behavior of nodes in the OAFSA algorithm. The more obstacles exist in the water, the more the OAFSA algorithm will fail and the lower the network coverage will be. When the obstacles are distributed in the water, they are random and may overlap with the position of the target monitoring points of the same random distribution. After a node meets an obstacle, it will miss the coverage of the target monitoring point that is close to the obstacle. These two points cause the RAWS algorithm’s coverage to be reduced by a small margin with the increase in the number of obstacles.

**Figure 9:**
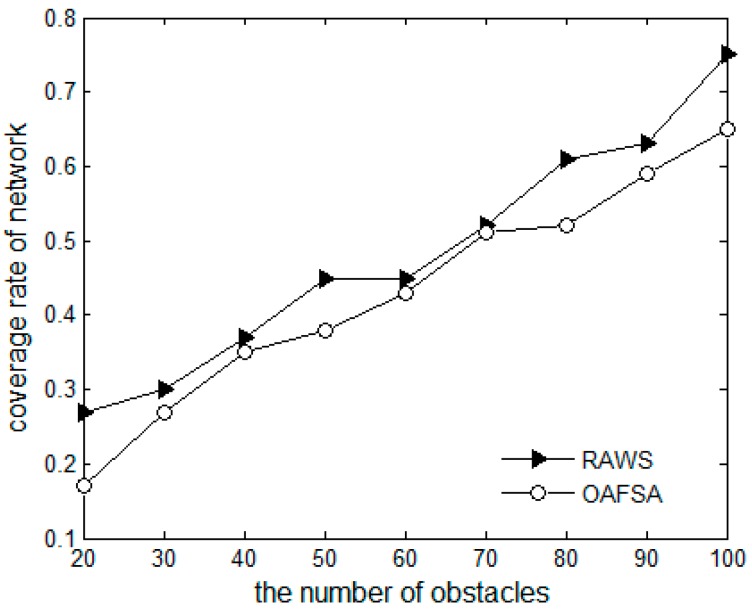
Comparison of network coverage rate at varying numbers of nodes.

Figure 9 illustrates the network coverage rate of the OAFSA and RAWS algorithms with the number of nodes in the network. As shown in the figure, with the increase in the number of nodes in the monitoring space, the coverage rate of the two algorithms increases. However, the RAWS algorithm always obtains a coverage rate that is higher than that of OAFSA algorithm because any two nodes will not move in the same direction in the RAWS algorithm unless a common goal exists. The RAWS algorithm can search multiple regions of the monitoring space without a wide range of information exchange. This capability ensures that nodes avoid local optimization, expands the scope of the node search, and improves the quality of coverage. In addition, the use of congestion control improves the coverage effect.

**Figure 10:**
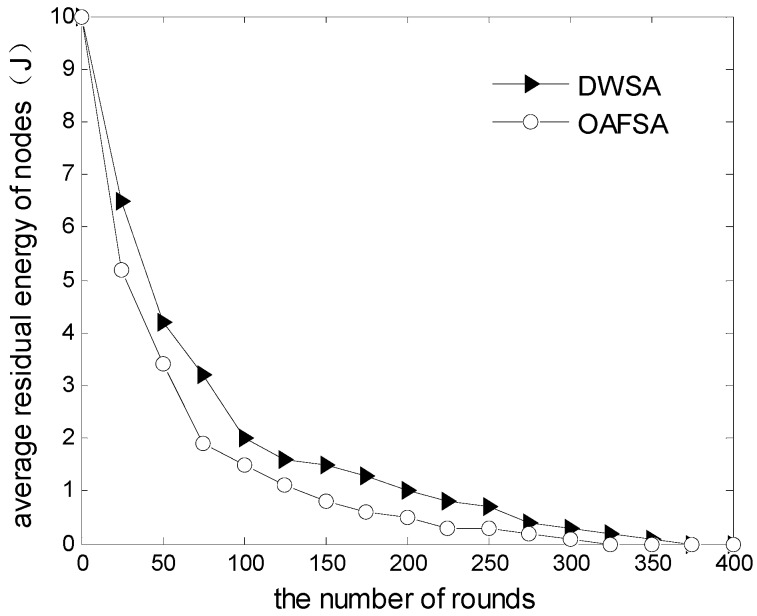
Comparison of average residual energy of nodes at varying numbers of nodes.

Figure 10 shows the change in network average residual energy with the network running round number. Because the RAWS algorithm uses an independent search method in the underwater moving process, information is transmitted only in the neighbor nodes. Thus, the average residual energy of nodes in the RAWS algorithm is generally higher than that of the OAFSA algorithm. The entire group does not need to interact. This condition significantly reduces the node energy consumption in the data transmission process and extends the entire network’s running time. This is one of the advantages of the improved wolf search algorithm over other intelligent algorithms.

**Figure 11:**
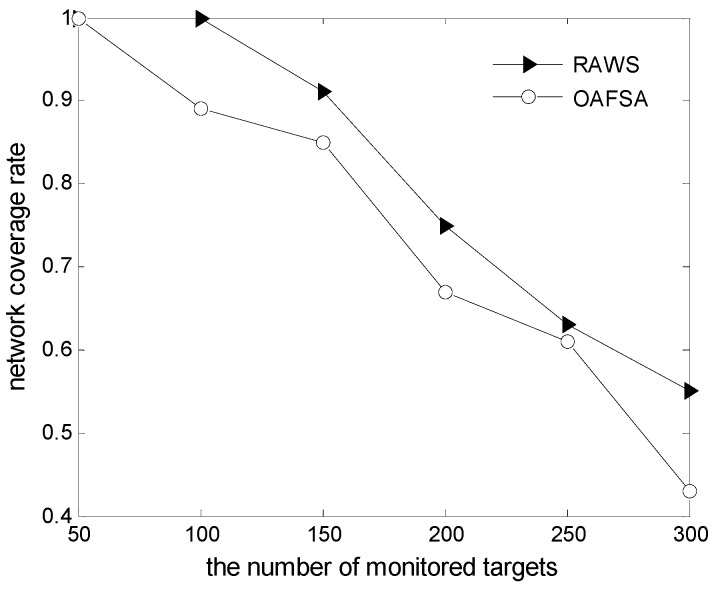
Comparison of average residual energy of nodes at varying numbers of monitored targets.

Figure 11 shows the change in a network coverage rate with the monitored targets’ number. Obviously, when the monitored targets’ number is 50, the two algorithms’ coverage rate is 1. It shows that all monitored targets are covered. When the monitored targets’ number is 100, the coverage rate of RAWS algorithm is one. The coverage rate of OAFSA algorithm is 0.89. It shows that all monitored targets are covered. It shows that some nodes are invalid in the OAFSA algorithm due to obstacles. Naturally, the coverage rate is lower with the increase of monitored targets in the OAFSA algorithm.

## 6. Conclusions

An underwater sensor network redeployment algorithm based on wolf search was developed to solve the problem of node deployment optimization with existing obstacles. In the process of node redeployment, the algorithm always exhibits implicit parallelism, and each node searches for the monitoring target independently without exchanging information with the other nodes. A number of areas of the monitoring space are searched. The algorithm has good coverage and low energy consumption. When congestion control is incorporated in the algorithm, it makes the node distribution tending to event distribution. When nodes encounter obstacles, they escape automatically. Hence, it prevents node failure caused by external force. Meanwhile, the algorithm jumps out of the local optimal solution. For our future work, because our obstacles are limited, we plan to establish different types of obstacles and distribution models that combine specific environmental factors, such as water flow. Experiments will then be conducted in a specific water environment.

## Figures and Tables

**Table 1:** Simulation parameters.

Parameter	Value
Sensing radius R (m)	30
Communication radius r (m)	60
Initial energy consumption Eint (J)	10
Carrier frequency f (KHZ)	25
Energy consumption of data reception Pr (mW)	3
Uncertainty factor Re (m)	15
Moving step length s (m)	0 < s < 30
Moving speed ∂ (m/s)	1
Relevant measuring equipment parameters α	0.2
Relevant measuring equipment parameters β	2
Obstacle number Obs	20
Common node number Nod	100
Energy diffusion factor η (Khz)	2
